# A way of relating to life; myself and others - a thematic analysis of patients’ experience of having an eating disorder

**DOI:** 10.1186/s40337-025-01291-1

**Published:** 2025-05-26

**Authors:** Malin Bäck, Sanna Aila Gustafsson, Karin Jacobson, Theresia Ljung, Rolf Holmqvist, Gerhard Andersson

**Affiliations:** 1 Department of Behavioural Sciences and Learning, Linköping University, Linköping, Sweden; 2 Futurum: Academy for Health and Care, Region Jönköping County, Värnamo, Sweden; 3 Faculty of Medicine and Health, University Health Care Research Center, Örebro University, Örebro, Sweden; 4 School of Behavioural, Social and Legal Sciences, Örebro University, Örebro, Sweden; 5 Department of Clinical Neuroscience, Karolinska Institute, Stockholm, Sweden

**Keywords:** Eating disorder, Patient perspective, Lived experience, Qualitative study, Ego-syntonicity, Self-observation, Externalization

## Abstract

**Background:**

Eating disorders are psychiatric conditions that extend beyond concerns with weight, body and shape, encompassing complex issues related to self-esteem, emotion regulation and interpersonal functioning. Moreover, co-occurring depression, often undiagnosed, is a common complicating factor. Gaining an in-depth understanding of living with an eating disorder is vital both theoretically and for identifying factors that maintain or inhibit recovery. Patient-centred studies offer valuable insights into the lived experience of eating disorders, highlighting their impact and interaction with various life phenomena.

**Methods:**

This qualitative study aimed to capture the meaning and experience of living with an eating disorder from a patient perspective. Semi-structured interviews were conducted with 15 women suffering from eating disorders and comorbid depressive symptoms, prior to the start of treatment. Data were analysed using reflexive thematic analysis.

**Results:**

Two dimensions of relating to the eating disorder emerged. The first dimension, “Relating to the eating disorder over time”, followed a temporal trajectory with four themes: “The eating disorder as a way to handle other difficulties”, “From control to a consistent loss of control”, “The whole existence revolves around the eating disorder” and “Hard to see a life without the eating disorder”. The second dimension, “Having an eating disorder– a relentless relating”, focused on the present experience and was divided into two themes: “The eating disorder’s impact on relating to myself” and “The eating disorder’s impact on relating to others”.

**Conclusions:**

Living with an eating disorder involves a constant, entangled and conflicted relationship with the disorder, leading to alienation from significant others and one’s own body. This pervasive presence of disordered thoughts and behaviours makes it challenging to relate to oneself and the external world without their influence. Over time, the disorder becomes increasingly ego-syntonic, rendering it difficult to envisage a life without it. Treatment should, therefore, aim to externalise the disorder—to foster new life goals, enhance social engagement, and improve interpersonal skills. Further research is needed to elucidate how co-occurring depressive symptomatology influences an individual’s relationship with their eating disorder, as these factors may be crucial in tailoring effective interventions.

**Supplementary Information:**

The online version contains supplementary material available at 10.1186/s40337-025-01291-1.

## Abstract (es)

To capture the meaning and experience of living with an eating disorder from the patient’s perspective, 15 women with eating disorders and depressive symptoms were interviewed before treatment. Reflexive thematic analysis was used to analyse the data. Two main dimensions of relating to the eating disorder were identified. The first dimension, “Having an eating disorder - a relentless relating,” described the present experience and included themes about how the disorder affects their relationships with themselves and others. The second dimension, “Relating to the eating disorder over time,” followed a timeline with themes showing how the disorder evolved from a coping strategy to a pervasive force affecting their entire existence. Living with an eating disorder means constantly experiencing a chaotic relationship with the disorder that distances individuals from others and their own bodies, making it challenging to imagine life without the disorder. Treatment should help individuals see the disorder as separate from themselves, reconnect with their bodies, and acknowledge their needs. It should also foster new life goals, social engagement, and improved interpersonal skills for a meaningful life beyond the eating disorder. Further research is needed to understand how depressive symptoms influence the experience of living with an eating disorder and the recovery process, as these factors may be crucial in tailoring effective interventions.

## Background

Studies have reported that living with an Eating disorder (ED) is not only about weight, body, and shape. It also involves difficulties in the fields of emotions, self-esteem, interpersonal and cognitive function [1]. EDs are a category of psychiatric disorders that have been studied from various theoretical perspectives [2] and are commonly regarded as multifactorial conditions [1, 3]. Furthermore, comorbidity with other psychiatric disorders, such as depression, is common but often underdiagnosed, and this comorbidity can sometimes be seen as an intrinsic part of the ED itself, where the bidirectional interaction between these conditions can intensify their symptoms and create additional challenges for recovery [4, 5].

Research has explored how certain vulnerability factors increase the risk of developing an ED and the problems that arise as a consequence due to the ED [6]. These interconnected factors shape the lived experience of EDs, impacting individuals’ self-perception and quality of life. Identifying the maintaining and hindering factors associated with EDs, regardless of their origins, can benefit from insights into patients’ subjective experiences. Research focusing on how EDs influence and are influenced by various life phenomena from the patient´s perspective is particularly relevant for complementing existing knowledge.

Previous qualitative studies have predominantly investigated the experiences of ED patients during or after ED treatment, with a primary focus on individuals suffering from Anorexia nervosa (AN) [7–9]. While these studies have provided valuable insights, they often focus on treatment processes and recovery from a retrospective perspective, leaving questions about the experiences of patients who are not currently engaged in ED treatment partially unanswered. A systematic synthesis of qualitative findings has examined the perspectives of patients, family members, and healthcare professionals regarding ED and ED treatment, highlighting significant differences in views on treatment approaches and expectations among these groups. It was emphasized that further research is needed on patients’ perspectives of living with different forms of EDs and on the factors that either facilitate or hinder recovery [10]. Regarding existing research on patients ‘experience of living with an ED, these studies suggest that EDs may be experienced as all-encompassing, with some patients describing living with the disorder as a form of internal coercion where the ED dictates their lives and impacts their relationships with loved ones [11, 12]. For instance, adolescents with AN have reported that their lives were put on hold during treatment and that relationships and social contexts played a pivotal role in recovery [13]. Similarly, women with Bulimia nervosa (BN) have described their ED as a secret internal battle, where ED behaviours were carried out to calm their inner selves. These behaviours were accompanied by secrecy, shame, and fear of judgment, which often led to isolation. For these individuals, the ED became a central aspect of their identity, and the prospect of living without it evoked fear [14]. In contrast, individuals with Binge eating disorder (BED) have likened their experience to a challenging addiction, describing it as living in a negative spiral where thoughts and emotions were the most inhibiting factors. These patients struggled with low self-esteem, a lack of understanding from others, and feelings of shame but found validation and relief in receiving a diagnosis [15].

Understanding the meaning of living with an ED on a deeper level is essential both from a theoretical perspective and clinical practice. It is particularly crucial to study the patient’s experience of living with an ED while it is ongoing, as this provides insight into its impact on their life, without the influence or bias of therapy. Such an approach allows for a more authentic understanding of the disorder as it is experienced by the patient in real time, offering valuable information for both treatment planning and theoretical development.

## Method

### Design

The aim of this qualitative study was to capture the meaning and the experience of living with an ED from a patient perspective. For the identification of themes related to the experience of having an ED, a reflexive thematic analysis was employed [16], based on interviews with patients who had been diagnosed with an ED and were awaiting the start of treatment.

### Participants

The participants in this study were 15 patients from Swedish ED services, awaiting treatment as part of a naturalistic study on Interpersonal Psychotherapy (IPT) [4, 17]. Inclusion criteria comprised both women and men aged 16 and above with a Body Mass Index (BMI) of at least 17; only women participated, likely due to the higher number of women seeking help for EDs. A total of 48 patients participated in the IPT study, with the first 25 being interviewed using the interview guide applied in the present study (see section Interview for further details). The patients had previously been assessed within the Swedish national patient register (NPR) framework [18] and were subsequently both interviewed and subjected to a more in-depth diagnostic assessment by their assigned therapists at their regular ED unit before therapy began. The therapists were trained in all instruments used in the study.

In addition to the Symptom-Specific Reflective Functioning Interview for Eating Disorders (SSRF-ED) [19], which provided the data analysed in this study, the following measures were used during the pre-assessment: The Eating Disorder Examination Questionnaire (EDE-Q), The Eating Disorder Inventory-3 (EDI-3), The Montgomery Åsberg Depression Rating Scale (MADRS), and the Experiences in Close Relationships scale (ECR) [4, 17]. The participants, aged 17–39 years, were mostly working or studying. While detailed data on previous treatment histories are not available for all participants, most were awaiting their first experience of ED-specific treatment. Based on these instruments, as well as clinical assessment, the interviewed participants exhibited a moderate to high level of ED symptoms, and depressive episodes ranged from mild to severe. At the time of the interviews, the relationship between the participants’ ED and depressive symptomatology remained unclear—whether depression co-occurred with the ED or stemmed from other factors. This relationship was further explored during therapy, as the treatment aimed to clarify the links between ongoing symptoms and interpersonal factors. Some participants had a known diagnosis of depression prior to inclusion, while for others, depressive symptomatology became evident only during the assessment process. Although this study does not focus on the experience of being depressed or investigate whether co-occurring depression influences the experience of having an ED, these patient data are relevant to mention, as depression is frequent, though often undiagnosed, in individuals with EDs [20, 21]. Furthermore, regarding the aim of the study and the focus of the thematic analysis, patients were not categorised into ED subgroups based on symptom profiles but were treated as a unified group. This aligns with Fairburn’s transdiagnostic model, which conceptualises ED’s as a phenomenon that manifests differently in individuals while sharing underlying mechanisms [1, 22]. However, the group was mixed, predominantly comprising individuals with BN or subthreshold conditions—specifically, those defined in DSM-5 as Other Specified Feeding or Eating Disorder (OSFED), characterised by a mixed presentation of AN and BN with transitional or combined symptomatology [23, 24].

### Interview

The data analysed in this study were derived from interviews conducted before the start of treatment in the previously mentioned study. The Symptom Specific Reflective Functioning interview for Eating Disorders (SSRF-ED) by Skårderud et al. 2012, comprises 13 questions focusing on the individual’s experience of the connection between the ED and thoughts, feelings, bodily sensations, situations, and relationships. The interview also includes questions about the ED’s function and impact on life [19, 25] (for further details, see supplementary material). SSRF-ED, is rooted in the mentalization tradition [26], and is an adaptation of the symptom specific reflective functioning interview (SSRF) [27, 28]. The interview was part of the study project’s comprehensive pre, mid, and post measurements with the intention of evaluating any changes in reflective functioning (RF) in relation to their ED during the therapy process. However, the purpose of this present study was not to rate RF. Nevertheless, a qualitative analysis of the interview responses in SSRF-ED was considered feasible in order to document the participants´ experience of having an ED. The questions in SSRF-ED and its semi-structured format makes it possible to capture relevant phenomena related to experiencing. However, the strength of this choice of data is that it is taken from a mixed group of individuals with EDs, and the interviews are conducted before the treatment begins. Considering that previous qualitative research mainly has examined experiences of ED treatment or views on the ED after recovery, we view this design as a relevant addition [29].

#### Selection of interviews

Of the interviews conducted, 15 met the criteria for sufficient technical quality, defined by clear audio, minimal noise, and detailed, relevant participant responses. During the analysis, conceptual depth was considered to have been reached after just over half of the interviews; however, we chose to include all 15 interviews in the analysis. The interviews lasted approximately 30 min, with two exceptions: one lasting 65 min and the other 15 min.

### Thematic analysis

In order to capture the informants’ subjective experiences of having an ED, the interviews were processed and analysed with Braun and Clarke’s model for reflexive thematic analysis (TA [16].

During the analytical process of the TA, four researchers closely collaborated, all of whom have training in qualitative research methodology. They also have extensive clinical experience in working with patients with EDs. However, none of the researchers were interviewers or therapists for the participants in this study. A description of how the collected data was processed and analysed is presented in Table 1.

**Table 1:** Process of reflexive thematic analysis

Step 1: Familiarization with the data	The 15 recorded interviews were reviewed multiple times and transcribed. During this process, notes were made on key aspects that stood out and an inductive approach was employed. The interviewer’s questions and comments then were removed from the transcripts, leaving only the patients´ description of their experiencing their ED for further analysis.
Step 2: Coding	Transcripts were carefully read through, and with a focus on the aim of the study, segments of the text were summarized into relevant codes that described the meaning of the data (semantic codes) Some latent codes were also identified at a more interpretive level. All codes were then compiled along with the data relevant to each code.
Step 3–5: Generating initial themes, developing, and reviewing themes, refining, defining and naming themes	Once the codes had been identified, the process of developing initial themes began. Similar codes were merged, and the merged codes were examined to identify commonalities. It became clear that there were several ways to thematize the data based on the codes. After further processing, initial themes were developed with the study´s purpose and research question in mind. All codes were then reviewed to assess how frequently they recurred within each theme. Relevant themes and sub-themes were then defined, named and described, and these were subsequently refined.
Step 6: Writing up	Writing the results section, which involved describing the themes and sub-themes and selecting appropriate quotes to illustrate each, provided a final opportunity to refine the analysis, including determining the most effective order in which to present the themes. Pseudonyms were used to protect participants’ identities, and to highlight the uniqueness of the individual behind each quote, their current symptom profile/diagnosis was specified.

## Results

**Fig. 1:**
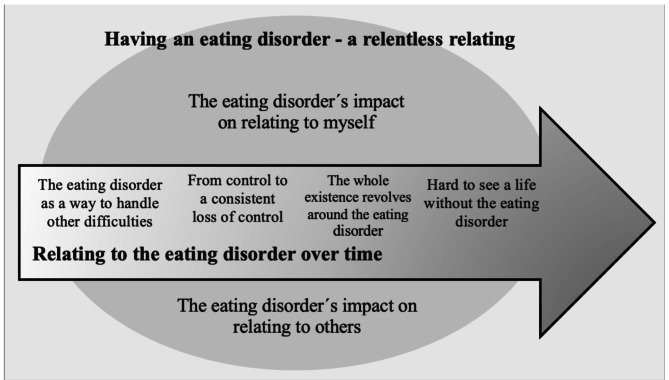
Two dimensions of the experience of living with an ED

The analysis of the participants’ experiences revealed *two interconnected dimensions of living with an ED*. The first theme was captured by the overarching concept of *Relating to the ED over time.* This dimension follows a chronological trajectory in which the informants’ experiences with the ED evolved, initially perceiving it as a coping strategy. However, over time, a shift occurred from a sense of control to a loss of control as the complexity of ED symptoms intensified. Ultimately, the ED came to dominate their entire existence, making it difficult to envision life without it. The second theme, *Having an ED– a relentless relating*, reflects the ongoing experience in the present moment. This dimension was divided into two parallel subthemes: Relating to oneself and Relating to others. These subthemes highlight the participants’ sense of entrapment in their relationships with themselves, others, and the ED. The ED was experienced as an ever-present force, to which they inevitably adapted and related. The results are summarized and illustrated in Fig. 1, with each theme and subtheme described in greater detail below.

### Relating to the ED over time

This dimension describes how the participants’ relationship to the ED changed over time, with the ED often initially being perceived as a solution to another problem in everyday life. Over time, however, the ED became more cemented, as a part of the person, creating uncertainty about what a life without the ED would be like. This contributed to an ambivalence about the path forward towards a healthy life without the ED. Based on these descriptions, we organised this theme as a timeline with four subthemes that progressively reflect how the ED becomes more entangled and complex in the individual’s experience over time.

#### The ED as a way to handle other difficulties

The onset of the ED was described in the context of external stressors, disputes, losses, or adjustments, that became hard to handle. ED behaviours emerged as a way to cope and were described as a temporary consolation, offering the informants their own safe space to control their body when other aspects in life felt out of their control.

*I fell into a depression*,* and*,* because of the circumstances*,* I imagined taking a little better care of myself by eating more regularly but yeah… And eventually it went overboard somehow and in retrospect I have figured that it became a way to eventually feel in control*,* a terrible control issue…which I applied to food and through workouts. It was not foremost about being unsatisfied with my body or it did not begin as a result of that… instead it was about control I presume. There was a lot of losses and pressure around me back then… (JENNY- BN*,* major depression)*.

The ED was described as something else to focus on, a comfort or a way to regulate emotions around problems in family or peer relations.

*Like later when I began*,* you know*,* like when it got too much for me emotionally in life*,* then it turned into me putting like*,* I had to put my feelings somewhere and then it turned into like an obsession with working out and focusing on my body. (SARA- BN*,* subthreshold depression)*

#### From control to a consistent loss of control

The initial experience of control seemed to change over time, as the pursuit of control became too demanding and inevitably led to a perceived loss of control. This increasing entanglement involved strict self-imposed standards and over-controlling behaviours gradually becoming integrated into the personality in an unreflective manner. Over time, this further reinforced the hold of the disorder.

*Yeah*,* it feels like you aren’t enough*,* whatever I do like*,* like yeah… This thing with me eating rigidly and working out a lot…it is a lot about this thing with control…*,* the fact that I want to keep track of what I do*,* and I want to see*,* yeah*,* simply put control myself. In some way… Yes*,* because then I feel… yes*,* but then I lose control and then I start binge eating. Because I don’t have the energy to stay in control for so long because it is wearing that as well. It just happens. (LYDIA -BN*,* mild depression)*

#### The whole existence revolves around the ED

The participants described how managing the ED became their primary concern, with school or work relegated to secondary tasks that also had to be taken care of. Being in a competitive or stressful occupational context often led to stricter, more dichotomous ED-rules. This created a vicious cycle, where the participants withdrew from important areas of life when they felt unable to meet their own standards, leading to their entire existence becoming controlled by the ED. Perfectionism, self-invalidating thoughts and rigid demands around body control, food, and performance were central, shaping both behaviour and identity.

*Well…when they are as strongest it is all about that*,* more or less*,* I mean it is*,* all about getting as perfect of a day as possible. Yeah*,* and everything else is a little bit in the way because I have to have it my way. And only my way. (LYDIA– BN*,* mild depression)*

#### Hard to see a life without the ED

It was difficult for the participants to imagine a life without the ED. However, they could describe moments of meaning and engaging contexts — both professionally and in their spare time — where they, for a while, shift their focus to other areas in life, such as spending time with loved ones, being out in nature, or focusing on something bigger than themselves.

*It makes things easier to have things to do that are not connected to the anxiety-triggering things. To maybe be outside on a long walk with the dogs or meeting a friend for a chat. (KAYA– BN*,* subthreshold depression)*

There was a longing to approach life with a broader perspective, and they felt that the ED impeded this goal. However, these moments were described as brief pauses, and the idea of what life would be like without the ED seemed hypothetical and incomprehensible. The participants’ thoughts about a life without being sick were concrete with reflections embedded in an ED mindset.

*Naah… hard. Can’t even imagine how that would be like… Would probably have been happier at least… (SARA– BN*,* subthreshold depression)*.

*It would be a lot*,* a lot better*,* I mean I know that. I mean I would definitely get involved in things a lot more than what I do now. And really become that outgoing person. I mean… Right now*,* it has been too little…*,* before I always let people in*,* I always wanted to help people*,* and I had all the energy and all that… and now I often feel that I do not have the energy for anyone because I can’t even cope with myself… kind of like this. So*,* it would be a lot more fun*,* much easier and much better. (SUE– BN*,* mild depression)*

### Having an eating disorder - a relentless relating

This dimension/theme describes having an ED as a *relentless relating* meaning that the participants’ emotions towards their ED were entangled in such a way that the ED was seen as a part of one’s own person rather than a condition. The ED was experienced as something ever-present, which they inevitably had to relate to in every situation. It was described as life not working without the ED, but at the same time, life not working because of the ED. We organized this dimension in two themes: the EDs impact on *relating to oneself* and the EDs impact on *relating to others*.

#### The EDs impact on relating to myself

The ED was described as something ego-syntonic, affecting every area in life. It was described as an obviously intertwined part of oneself, like a matter of course. The ED became a way of understanding and taking care of oneself, albeit in a harsh and judgmental way. The informants appeared to find it difficult to reflect on themselves without filtering themselves through the lens of the ED. This way of relating seemed to make it challenging to be close to oneself and one´s body. In line with this, the patients reported an invading closeness to their ED thoughts — which were experienced as self-evident and unquestioned — while simultaneously feeling a dismissive distance to their own inner states, in a way that invalidated their emotional needs and sense of self.

*It is such a big part of how I feel (…) I feel so much better if I am comfortable with my body. If the scale has showed a number that is fine like*,* or yeah*,* isn’t that high*,* then I like myself a lot more and feel so much better and am so much more satisfied… the entire day can be impacted. (…) About feelings it is like*,* I don’t really know which affects which… if it is the fact I feel fat that makes me sad or the other way around*,* but if I feel depressed or angry or sad or worried or something like that*,* then I often feel bigger*,* often feeling big and fat. It turns into not just me feeling miserable*,* I´m fat and ugly too. (MARY -OFSED*,* subthreshold depression)*

Although the ED had a significant negative impact on life, ED behaviours were also described as helpful in creating a sense of control, and as a way to deal with emotions and other inner sensations. Restricting oneself, bingeing and purging were described as calming and as a way to regulate various difficult experiences.

*There are quite a lot of different thoughts and feelings. Maybe the inability to feel grounded in myself and notice… now I am mad*,* now I am mad at this person… or becoming sad or becoming tired or*,* daring to figure out those feelings. There are quite a lot of feelings being suppressed. (…) The food then becomes an escape*,* a pleasure and yeah*,* a refuge*,* I guess. (JENNY - BN*,* major depression)*

However, the ED was not only used to regulate specific negative affects in specific situations, but it was also often described as an automated and natural part of regulating oneself in general.

So, like when I ate, then I got a kick out if it, but of the opposite kind. And I guess I am still doing that, mostly, when I am,…it could be when I am happy as well… not just when I’m feeling down… but when it turns into strong emotions then, like… then it turns into me wanting to calm myself by eating. (*TILLY– BN*,* moderate depressive episode)*

In contrast to the entanglement between the individual’s experience of herself and the ED, the participants described their own bodies as something ego-dystonic and external to themselves — something that they were forced to live with. The body was seen as separate from oneself, an object to invalidate and to judge för letting them down. The informants described their bodies as disobedient, needing to be controlled and regulated.

*Not that good*,* I… I think about it very often*,* I mean myself and the body*,* like in two parts. I mean it is something that I*,*. No but it is like my persona*,* my personality or my ego. and then it is like the body…that I am forced to live with. Yes*,* I feel like that often*,* like it is always me against my body. (TILLY– BN*,* moderate depressive episode)*

This way of relating seemed to make it hard to be in contact with one´s body. Inner experiences such as vulnerability were described as concrete bodily sensations like heaviness or lightness. The experience of one’s own body could be linked to the individual’s mood and emotional experiences. For example, this could manifest as the body feeling like it is swelling uncontrollably or growing, difficulties in moving into different positions due to how the body feels, and a sense of pain in different parts of the body that the person is dissatisfied with. These experiences were typically described like something to dismiss and disregard.

*When the body feels unpleasant*,* I mean when you feel. it is almost as if*,* someone is sitting and pinching*,* I mean like almost phantom pain*,* someone sitting and pinching the back of the thigh or on the butt or at the belly. (LOU– BN*,* major depression)*

A great discomfort was described when others touched them, stemming from the individual’s own body dissatisfaction. This discomfort created difficulties with physical contact and intimacy, as the negative perception of their body acted as a barrier not only to self-acceptance but also to forming connections with others. Body dissatisfaction was therefore described as a significant obstacle in forming relationships, leading to increased isolation. The impact of the ED on the ability to relate to and be close to oneself was described as contributing to these difficulties, as the inability to connect with oneself made it challenging to experience intimacy with others.

#### The EDs impact on relating to others

Having an ED profoundly influenced the individual’s social interactions and relationships with others to a great extent. The ED was described almost like a harsh partner in a destructive relationship. Each situation needed to be adapted to the requirements of the ED and often the participant chose to refrain from activities with other people in order to avoid lying or compromising with rules linked to the ED. The participants felt compelled to defend their ED behaviours in relation to loved ones, which could lead to open or hidden conflicts with important others.

*I mean I have chosen to end quite a few relationships and it is possibly because of this I can’t decide everything when someone else is involved*,* like what to do*,* what to eat*,* when to eat*,* activities*,* and stuff like that You have to adapt to others… so I have consciously made these choices to keep relationships short and superficial*,* because I don’t know how I’d react if things got more serious or lasted longer. It’s also these feelings of either someone else breaking it off or me doing it*,* so it’s better if I do it myself*,* because I don’t know how I’ll feel down the road about my body or food. (CAREN– OFSED*,* moderate depressive episode)*

This often led to withdrawal and isolation in the long run. The informants described an ambivalence regarding their relational needs to others and expressed feelings of loneliness, even when in the company of others. They also felt that relatives were unable to fully understand the complexity of having an ED. Although the participants chose to isolate themselves. They also expressed a fear of being alone, particularly as they worried about losing control over the urge to binge.

*When I am alone it generally gets worse in every way. Either I don’t eat anything and eat very restrictively when I am alone and trying to avoid all types of food I mean. Or if I am alone*,* it can also turn the opposite way*,* I might binge eating or yeah*,* eat and throw up and eat normal-sized portions as well and throw up. And then when it comes to situations with dinners and stuff like that with people*,* then it is usually the case that I eat but then go and throw up afterwards because I want to in some kind of way show that there aren’t any issues and that I can eat this. (MARY– OFSED*,* moderate depressive episode)*

The ED was described as *the big secret* that they were keen to keep hidden. They wanted to be able to talk more openly with loved ones about how they felt, but this was hindered by fear that they would be hindered from engaging in ED behaviours if they were to let other people in. These contradictory desires became an exhausting vicious circle that led to an emotional loneliness and isolation.

*I don’t think that (that) many people believe I am this unhappy with who I am*,* I have two sides. The “outside” with others*,* where you are. where everything goes very well and there aren’t any problems and like that. So*,* I think a lot of people would be like*,* oh*,* or like are you that unhappy*,* are you like that…… (LOU– BN*,* major depression)*.

Relationships with others were also complicated by exhausting social comparisons regarding appearance or achievements. The participants’ own notion of what others might think of them were filtered through their demands for perfection and adherence to food rules — a kind of *self*-*invalidation by proxy*. Comments from others were often interpreted as critical or demanding, influenced by the participants´ own high expectations of themselves. This made it difficult to relate to others for support and contributed to the feelings of loneliness.

*It becomes…. I mean I don’t think they actually put that much pressure on me but to me*,* like me*,* where I am at the moment it feels like quite a lot and then it very often like*,* like it triggers it. And then I have some*,* or mostly one friend who has had an ED themselves but not gotten help with it so with her especially I feel like we trigger each other quite a lot. (TILLY– BN*,* moderate depressive episode)*

Another factor that made it difficult to lean on others for support was the desire to protect those around them from fully understanding the true nature of the ED. This created an unfortunate vicious cycle, where withdrawing from others led to loneliness, which in turn made the participants even more dependent on having the ED to turn to.

## Discussion

The aim of this study was to capture the inner experience of living with an ED from a patient perspective. The results revealed two overlapping dimensions of relating to the disorder: one capturing the direct, moment-to-moment experience of the ED, and the other reflecting a more processual perspective, in which the ED gradually becomes ingrained in the individual’s self-concept and way of navigating life. These dimensions are not separate but intersect, illustrating how individuals experience the ED both as an immediate, lived reality and as a structure shaping their identity and relational patterns over time. The identified themes could broadly be understood as an unconditional relating to the ED—something that can be described as “a relentless relating.” Individuals carry the ED with them constantly—initially as a coping mechanism for burdensome aspects of life, but over time, it evolves into something increasingly intricate, yet simultaneously inherent and difficult to imagine life without. This shift reflects the processual dimension, where the ED transforms from a tool for managing distress into a rigid and consuming force that structures the individual’s entire existence. At the same time, in their immediate experience, participants described a deep entanglement between ED-related thoughts and their sense of self. The ED was perceived as an internalized presence—comparable to an inner voice or a harmful relationship—that left little room for interpersonal connections. This ambivalence was evident in the participants’ simultaneous longing for closeness and understanding from loved ones while also experiencing shame and a need to conceal the ED, leading to withdrawal or a sense of living a double life.

### The ED as an integrated part of oneself

The finding that the ED becomes increasingly ego-syntonic over time, as an integrating into the individual´s sense of self to the extent that they cannot speak about themselves, their self-perception, or their emotions without filtering them through the ED, aligns with previous research, primarily conducted with AN patients. Such research describes the ED as an inner voice, part of the identity, or as a different side of the self [30–32]. Studies on various ED conditions, including BN, AN, and BED, have demonstrated similar findings, although the inner voice phenomenon has been most extensively studied in AN [33]. There is a notion that individuals with BN or BED may be more likely to describe their ED as ego-dystonic [34]. However, our study did not find this to be the case. Instead, the theme of the ED as an integral part of the self was consistent across participants, regardless of whether they had BN, BED, or AN with subsequent bulimic behaviours. One speculative aspect is whether the participants’ entangled relationship with their ED is influenced by ongoing depressive symptoms. Depression has been repeatedly shown to impair mentalization abilities [35, 36], and given the high comorbidity between depression and EDs, the degree of depressive symptomatology could potentially affect how the individual experiences their ED. This may help explain why mood disorders, alongside other comorbid conditions, often need to be addressed before conventional ED treatments can be effective [1]. Other comorbid conditions, such as neuropsychiatric diagnoses and personality disorders, have also been shown to impair mentalization abilities. These conditions, when present alongside an ED, may further complicate the individual’s relationship with their disorder. However, this study primarily focused on the experience of living with an ED, without a specific emphasis on comorbidity during the analysis. The experience of symptoms being perceived as an integral part of oneself has also been studied in other conditions, such as suicidal thoughts in depression [37], anxiety disorders, and substance abuse [38, 39]. However, ego-syntonic, and ego-dystonic experiences are primarily described in EDs and Obsessive-compulsive disorders (OCD) [40, 41], where symptoms are simultaneously experienced as both a source of significant suffering and a means of coping with distress. This contradictory relationship is commonly found in compulsive conditions characterized by loss of control, obsession, and affective intolerance [41]. Further comparative studies across psychiatric conditions are recommended to explore these dynamics.

### A harmful relationship with no place for others

Due to the coercive nature of the inner voice and its impact on self- esteem, patients’ relationship with their ED has, in previous research, been compared to a relationship with elements of physical and emotional abuse, leaving no space for a life outside of the relationship with the ED relationship and forcing the individual into social withdrawal [42]. Forsén Mantilla et al. (2018) showed that comprehensible relationship patterns between patients and their ED emerged like a real-life relationship. Patients in that study tended to experience their ED as controlling and domineering. These patterns were found to hold true regardless of the ED diagnosis [43]. Despite being harsh and critical, patients often experience a sense of affiliation towards their ED [30]. Similar to our findings, the ED has been described as a helper in dealing with and avoiding negative emotions, providing a sense of safety and protection [32]. Regardless of whether this integrated part of oneself is dominating, demanding, or motivating, patients have been able to attach positive value to their symptoms, which are consistent with their needs, goals, and ideals. This duality could help explain why patients are often ambivalent about change and recovery. However, it has been shown that the more ego-syntonic thoughts about eating, and weight-related behaviours became, the more controlling and guiding the symptoms became [41]. Despite negative consequences, such as losing relationships with significant others and sacrificing other aspects of life, previous findings indicate an unconditional need to remain close to the ED in an almost symbiotic manner [44]. This aligns well with the findings of our study, which indicate that the relationship with the ED not only affects the individual’s relationship with themselves but also their interactions with significant others and social environments. Interactions with family members and friends become strained, with others perceived as threats to or even competitors with the relationship to the ED [45].

### The entanglement with the ED makes it difficult to be part of a relational context

Similar to the participants in this study, Levine (2012) highlighted how individuals with EDs may struggle to relate to others when they strongly identify with their ED, although the participants in our study expressed an ambivalent longing for understanding and intimacy [46]. Additionally, Bachner-Melman et al. (2023) discussed the difficulties individuals with EDs face in developing mutuality in relationships, as they tend to either overly satisfy the needs of family members and friends or meet the needs of the ED [47]. In a meta-analysis by Cozi and Ostuzzi [48], relational competence among individuals with EDs was examined. The results demonstrated an increased challenge for ED patients in developing self-agency and relating to others. Various ED diagnoses tended to exhibit different relational difficulties, some with ambivalence in relationships or complete withdrawal from oneself and others [48]. As a consequence of the ED, conflicts with significant others, as well as other interpersonal vulnerabilities such as loneliness, have been highlighted in research on individuals with EDs [46]. In the review by Levine (2012), loneliness was accompanied by both intra- and interpersonal conflicts, which manifested somewhat differently across various ED diagnoses. The findings underscore the importance of helping individuals with EDs in developing healthy relationships [46].

### The body as something ego-dystonic

In contrast to the ego-syntonic entanglement that our study highlights between the patient’s perception of themselves and their illness, a division between the self and the body is described, where patients filter their experiences of themselves, their needs, and their well-being through invalidating disordered thoughts, which serve as the guide for what is right and wrong. Participants’ way of discussing their relationship with their own bodies was detached and fragmented, and it can be likened to so-called somatoform dissociation, something that has been identified in individuals with ED in previous studies [49, 50]. It has been suggested that individuals with EDs use their bodies to solve various types of emotional and relational problems — a phenomenon referred to as concretism, linked to difficulties in understanding their own emotional states [51–53]. In our study, participants also mention ED behaviours and body contempt as ways to cope with life events that evoke emotions– the internal is made external, and emotional words become physical sensations, for example, *feeling light when one is happy*. This aligns with Skårderud and Fonagy’s (2012) description of how ED patients experience their bodies and emotions as unreal, as if they do not belong to them, often leading to feelings of emptiness and meaninglessness [54].

### Clinical implications

From a clinical perspective, the results of this study show that ED treatment may primarily need to focus on the individual’s ability to see their symptoms from the outside and themselves with their feelings and needs from within. The ego-syntonic state of the ED, the vicious bond between the individual and their ED, and the dissociation towards one’s body, could possibly explain the ambivalence in relation to change. Both treatment providers and loved ones may find it difficult to reach the patient, as the impact of the ED often disrupts communication and mutual understanding. It is difficult to work on one’s relationship with oneself and others or be ready for behavioural change without first breaking the magic of the ED. This may be particularly challenging when the patient is also experiencing depressive symptoms, as depression can intensify feelings of hopelessness and self-blame, further reinforcing their bond with the ED. ED symptoms need to be clarified as manifestations of a condition in an individualized and validating manner [55], as symptoms and behaviours that do not define the person, but rather represent an illness that can be overcome [1, 4]. One way to make ED behaviours and ED thoughts more ego-dystonic is through externalization, which can be done in various ways across treatment modalities. The aim of externalizing a psychiatric disorder is to help patients perceive the disorder as something separate, rather than as a part of the self, but as an illness that has great negative influence on life and requires treatment. Viewing the ED externally can help patients to accept treatment [31]. However, previous research does not solely indicate externalization as helpful; it can be experienced as invalidating, with the patient not feeling seen as a person or being taken seriously unless it is based on the patient’s current symptomatology and its unique relation to their ED [56]. By creating a gap between the disordered thoughts and the individual’s experience of what they feel and desire, the patient can access mentalization, affect awareness, and what they desire in life together with others. Björk and Ahlström (2008) highlighted how recovered patients no longer perceived the ED as an integrated part of themselves. They expressed a desire to engage in life in terms of social contexts, life goals, and relationships [57]. However, a crucial and challenging step is to open oneself up to recovery, allowing for the possibility of letting go of the ED. It has taken up most of the patient’s life, crowding out relationships, activities, and various developmental opportunities for many years. This can be a grieving process, where the patient may need to find alternatives to turn to in order not to experience abandonment or go on the impulse to return to the ED in need of support [32].

An important aspect in ED treatment may be to motivate and support the patient in reorienting themselves in life and to recognize which relationships and social contexts are beneficial for their recovery [13]. A systematic review by Leonidas and Dos Santos (2014) examined the importance of social support in recovering from an ED, arguing that treatment should focus on the relational aspects of EDs rather than solely the eating-related symptoms [58]. It has been suggested that learning to accept oneself and manage conflicted relationships is an important aspect of recovery [59]. In our study, participants expressed experiencing relief from their ED when engaging in meaningful activities with others. However, they also reported feeling a distance or losing relationships due to their ED. Participants in our study at times compare the ED to a harmful relationship. Previous research, which likens the relationship with the ED to that of a significant other, suggests that ED treatment should encourage patients to rebel against their ED [55]. From this perspective, important factors in breaking up with an ED may include increasing one´s sense of agency and self-validation, as well as building social support, leaning on family and friends, and moving forward.

### Strength and limitations

A strength of this study is that it provides an in-depth examination of patients’ perspectives on their experience of living with an ED. Similar studies have primarily been conducted post-treatment and have mainly focused on patients’ experiences of undergoing treatment, and recovering from their ED, and have predominantly involved patients with AN. In contrast, our interviews were conducted before the start of psychotherapy centring on the patients’ experiences of the illness itself. Furthermore, the participants in our study represent various types of EDs, at different stages, with co-occurring depression. In these aspects, the study addresses areas within the field where a knowledge gap remains.

There are both strengths and limitations to the fact that this study derives from a larger naturalistic study. A strength is that the included participants are those who seek treatment within the regular ED services, meaning that the potential diversity within the sample may be representative of patients who ultimately seek care for their ED in Sweden. However, a limitation is that our thematic analysis (TA) and research question are not entirely independent of the potential comorbidities among the participants. As part of the study design in the larger naturalistic study, aimed at addressing other research questions, we assessed participants for depression and unexpectedly found that all exhibited varying degrees of depressive symptomatology. However, the presence of depression was not a focus of this qualitative analysis. In the light of our findings, and a reflection on existing patient data, we have subsequently considered whether ongoing depressive symptoms may have influenced our results. This may constitute a finding in itself—one that needs to be highlighted—even though it cannot be regarded as part of the TA. At the same time, the themes identified in the analysis were consistent across participants, regardless of ED diagnosis or severity of depressive symptoms. This could also be considered a strength, as it raises an important research question: how does concurrent depression shape the individual’s relationship with their ED, and how might this influence their ability to recover? One hypothesis regarding this comorbidity, which we did not consider extensively in the interviews, is the general impairment in mentalizing ability during ongoing depression [27, 60]. Mentalizing capacity can also be temporarily affected in other conditions, such as anxiety disorders. However, studies, including Millrod’s work on panic disorder [61], suggest that in anxiety disorders, the impairment in mentalizing is more closely related to the specific symptoms of anxiety (e.g., panic) rather than a general decline in mentalizing ability, as is typically seen in depression. This distinction may be relevant when considering how mentalizing is impacted in EDs compared to depression. Having a high degree of depressive symptoms alongside the ED could potentially amplify the findings we present in the results section regarding difficulties in relating to others and self-reflection. This, in turn, highlights the importance of addressing the patient’s ongoing depression and relational issues with themselves and others. This, in turn, highlights the importance of addressing the patient’s ongoing depression and relational issues with themselves and others. However, this remains speculative, and we do not have data to determine how this may vary among our participants.

Furthermore, regarding the representativeness and generalisability of this study’s results, additional limitations exist. The participants consisted of 15 patients, all of whom were Western women, which limits the transferability of the findings to men or individuals with EDs from other parts of the world. More studies on patients’ experiences, unaffected by the healthcare system’s perspectives on the illness and its solutions, need to be conducted, preferably from a transcultural perspective.

We, the authors, and researchers of this study, are all Western psychotherapists with varying levels of experience working with ED patients. We have substantial theoretical and clinical knowledge of the psychological phenomena discussed in ED research. We are aware that this preunderstanding influences how we interpret and assimilate the data. As explained in the methodology section, we consciously moved between a semantic, inductive analysis, where the content stands on its own with a focus on what the participant says, and a more deductive analysis, focusing on how the participant describes their experience, involving more reflexive interpretations and capturing themes on a more latent level. Moreover, an interview guide primarily designed to measure reflective functioning (RF) in relation to current symptoms, the SSRF-ED, was used. The interview highlights different areas that may be affected by ED in various ways. Although the purpose of our study was not to assess participants’ ability to reflect on their ED, but rather to capture the experience and impact across different life areas, one cannot rule out that participants’ responses were influenced by the original content of the interview guide. Nor can we exclude the possibility that as clinical researchers, our findings may have been influenced by the interview guide’s focus on studying the concept of mentalization. A strength in this context is that the coders in the TA were not the same individuals as the interviewers, and that the interviewers’ questions and comments were not included in the transcripts used for coding.

## Conclusions

The experience of living with an ED is described as a constant presence, akin to an entangled and disorganized relationship, creating a sense of alienation both from significant others and from one’s own body. It becomes challenging to relate to oneself and the external world without the filter of ED thoughts and ED behaviours. Over time, regardless of the specific ED diagnosis, the disorder becomes increasingly ego-syntonic, making it difficult to imagine life without it. Given that the ED has become the central focus around which their lives revolve, treatment should aim to externalize the disorder, making it ego-dystonic, in order to foster the development of new life goals, encourage social engagement, improve interpersonal skills, and fill life with meaningful content beyond the ED. In addition, further research is needed to better understand how co-occurring difficulties, such as depression, influence an individual’s relationship with their ED. Exploring how these factors interact may be crucial in tailoring interventions that effectively address not only the ED itself but also the broader psychological challenges that shape the recovery process.

## Electronic supplementary material

Below is the link to the electronic supplementary material.

Supplementary Material 1

## Data Availability

The datasets used and/or analysed during the current study are available from the corresponding author on reasonable request.

## Abbreviations

AN: Anorexia nervosa
BED: Binge eating disorder
BN: Bulimia nervosa
DSM: 5–Diagnostic Statistical Manual of Mental Disorders
ED: Eating disorder
IPT: Interpersonal psychotherapy
NPR: Swedish national patient register
OCD: Obsessive compulsive disorders
OFSED: Other Specified Feeding or Eating Disorder
RF: Reflective functioning
SSRF: Symptom specific reflective functioning
SSRF: ED–Symptom specific reflective functioning eating disorders
TA: Thematic analysis
