# Supplementary material for: A way of relating to life; myself and others - a thematic analysis of patients’ experience of having an eating disorder
Source: J Eat Disord. 2025 May 26;13:88. doi: 10.1186/s40337-025-01291-1 (PMC12105121; doi:10.1186/s40337-025-01291-1)
Supplement: Supplementary file 1 — Supplementary Material 1 [file 40337_2025_1291_MOESM1_ESM.pdf]

Mentalizing and Eating Disorders

Reflective Function in Individuals with Eating Disorders

Purpose

In this interview, individuals suffering from eating disorders participate to provide statements that illustrate their understanding of possible relationships between symptoms, behaviour, and inner experiences. We aim to highlight both symptoms in the form of concrete behaviours—such as self-starvation, binge eating, and purging—and thoughts related to emotions and bodily experiences associated with eating disorders.

Background

This interview is theoretically grounded in the mentalization tradition. Fonagy and colleagues developed the Reflective Functioning Scale (RFS) to measure the ability to mentalize thoughts, emotions, and intentions in relation to both oneself and others (Fonagy et al., 1998). Reflective function (RF) can be reliably assessed using the Adult Attachment Interview (AAI) (Main & Goldwyn, 1998), which was later adapted into a shorter version, the Brief Reflective Functioning Interview (BRFI) (Rudden, Milrod & Target, 2005). Rutimann and Meehan (2012) demonstrated that this shorter version provides a reliable, valid, and time-efficient way to code reflective function.

Additionally, shorter interviews have been developed to assess reflective abilities in relation to specific symptoms. An example of this is Rudden and colleagues' instrument for symptom-specific reflective function in panic disorder (Rudden et al., 2006). One research question is whether differences exist between general reflective function and symptom-specific reflective function.

This interview, designed to assess reflective abilities in relation to eating disorders, builds on this entire tradition, with particular emphasis on the latter instrument developed for panic disorder.

References

Fonagy, P., Target, M., Steele, H., & Steele, M. (1998). Reflective-Functioning Manual Version 5: For application to Adult Attachment Interviews. Unpublished manuscript. University College London.

Main, M., & Goldwyn, R. (1998). Adult Attachment Classification System. Unpublished manuscript. University of California, Berkeley.

Rudden, M. G., Milrod, B., Target, M., Ackerman, S., & Graf, E. (2006). Reflective functioning in panic disorder patients: A pilot study. Journal of the American Psychoanalytic Association, 54, 1339-1343.

Rudden, M. G., Milrod, B., & Target, M. (2005). The Brief Reflective Functioning Interview. New York: Weill Cornell Medical College.

Rutimann, D. D., & Meehan, K. B. (2012). Validity of a brief interview for assessing reflective function. Journal of the American Psychoanalytic Association, 60, 577-589.

Conducting the Interview

Explanation for the Interviewee

In this interview, I am interested in hearing your reflections on how you understand your eating disorder and how it affects your life. I will be asking some questions where I have chosen to use the term “eating disorder.” Please let me know if there is a term that you feel better describes your experience and that you would prefer me to use. Eating disorder is a broad term that includes behaviours such as self-starvation, binge eating, and purging through vomiting,

excessive physical activity, or the use of medication, among others. It also encompasses thoughts, emotions, and experiences related to food, body, and weight.
Please let me know if anything is unclear. Thank you for your participation.

The Interview

1. **Why do you think you have an eating disorder?**
   – Do you see any connection between your difficulties and your relationship with your parents, family, and childhood?
2. **Have you ever noticed a connection between symptoms related to your eating disorder and your thoughts and feelings?**
   – If yes, what thoughts and feelings?
   – If yes, do you have any idea how these thoughts and feelings relate to your ED symptoms?
   – If no, can you identify any patterns of improvement or worsening of your ED symptoms?
3. **Have you ever noticed a connection between your ED symptoms and certain situations?**
   Examples of situations could include being alone, at school, at work, at home, or during spare time.
   – If yes, in which situations?
   – If yes, do you have any idea how these situations may be linked to your ED symptoms?
   – If no, proceed to the next question.
4. **Have you ever noticed a connection between your ED symptoms and the people you spend time with?**
   – If yes, which people?
   – If yes, do you have any idea how these people may influence your ED symptoms?
   – If no, proceed to the next question.
5. **Now, I would like to hear about your experiences of your own body. These questions are similar to the ones I have already asked:**
   – What is your relationship with your body?
   – Is there a connection between how satisfied or dissatisfied you are with your body and your emotional state, situations, and/or people?
   – If yes, please elaborate.
   – Is there a connection between how you experience your body and your emotional state, situations, and/or people?
   – If yes, please elaborate.
6. **Summarising what we have discussed so far, can you give one or more concrete examples of situations where your ED symptoms are least noticeable?**
   Alternatively: When do you think the least about issues related to food, weight, and body?
7. **Can you give one or more concrete examples of situations where your ED symptoms are most pronounced?**
   Alternatively: When do you think the most about issues related to food, weight, and body?
   – In what way are your thoughts, feelings, and bodily experiences different when your ED symptoms are at their strongest compared to when they are at their weakest?

8. **What impact has your eating disorder had on your life and on the person you are today?**
9. **Do you have any thoughts about whether your ED has served a specific function in your life?**
   – Possible positive functions?
   – Possible negative functions?
10. **Do you have any idea how your life might have been without your struggles with food, weight, and body?**
11. **Have your thoughts on why you have an eating disorder changed over time?**
12. **What significance has your eating disorder had for other people in your life?**
13. **Would any of your closest ones who are aware of your struggles have a different understanding or perception of your eating disorder than the one you have described in this interview?**
    – If no one is aware of your struggles: If they knew about your ED, would they have a different understanding or perception than the one you have described in this interview?

Adapted from Skårderud, Sommerfeldt & Lech, 2012 © Version 5

Skårderud F, Sommerfeldt B, Lech B. Mentalization and Eating Disorders -Reflective Function in Individuals with Eating Disorders, version 5. 2012.

Wallin A, Samuelsson K. Symptom specific reflective functioning for eating disorders: associations with affect consciousness, self-reported attachment style and eating disorder symptoms. http://liu.diva-portal.org/smash/record.jsf?pid=diva2%3A627784: Linköping University; 2013.
